# Epidemiological and clinical use of GMHAT-PC (Global Mental Health assessment tool – primary care) in cardiac patients

**DOI:** 10.1186/1745-0179-5-7

**Published:** 2009-04-13

**Authors:** Murali Krishna, Peter Lepping, Vimal K Sharma, John RM Copeland, Lorraine Lockwood, Margaret Williams

**Affiliations:** 1 Department of Psychiatry, Wrexham Maelor Hospital, North Wales NHS Trust, Wrexham, UK; 2 Department of Psychiatry, University of Liverpool, Liverpool, UK; 3 Cardiac rehabilitation services, Wrexham Maelor Hospital, North Wales NHS Trust, Wrexham, UK

## Abstract

**Background:**

A computer assisted interview, the GMHAT/PC has been developed to assist General Practitioners and other Health Professionals to make a quick, convenient and comprehensive standardised mental health assessment. It has proved to be a reliable and valid tool in our previous studies involving General Practitioners and Nurses. Little is known about its use in cardiac rehabilitation settings.

**Aim:**

The study aims to assess the feasibility of using a computer assisted diagnostic interview by nurses for patients attending Cardiac Rehabilitation Clinics and to examine the level of agreement between the GMHAT/PC diagnosis and a Psychiatrist clinical diagnosis. Prevalence of mental illness was also measured.

**Design:**

Cross sectional validation and feasibility study.

**Methods:**

Nurses using GMHAT/PC examined consecutive patients presenting to a cardiac rehabilitation centre. A total of 118 patients were assessed by nurses and consultant psychiatrist in cardiac rehabilitation centres. The kappa coefficient (κ), sensitivity, and specificity of the GMHAT/PC diagnosis were analysed as measures of validity. The time taken for the interview as well as feedback from patients and interviewers were indicators of feasibility. Data on prevalence of mental disorders in an outpatient cardiac rehabilitation setting was collected.

**Results:**

The mean duration of the interview was 14 minutes. Feedback from patients and interviewers indicated good practical feasibility. The agreement between GMHAT/PC interview-based diagnoses and consultant psychiatrists' ICD-10 criteria-based clinical diagnosis was good or excellent (κ = 0.76, sensitivity = 0.73, specificity = 0.90). The prevalence of mental disorders in this group was 22%, predominantly depression. Very few cases were on treatment.

**Conclusion:**

GMHAT/PC can assist nurses in making accurate mental health assessments and diagnoses in a cardiac rehabilitation setting and is acceptable to cardiac patients. It can successfully be used to gather epidemiological data and help in managing mental health problems in this group of patients.

## Background

Mental health problems are represented with three diagnoses (depression, schizophrenia and bipolar-affective disorder) amongst the ten leading causes of disability worldwide [1]. The prevalence of depression in cardiac diseases has been estimated at around 15% [2,3]. Though less well studied the prevalence of generalised anxiety disorder has been estimated in the range of 7 to 24% in cardiac outpatients [4,5]. But there is a dearth of studies looking at a full range of psychiatric disorders in this patient group. The twelve-month prevalence and odds of major depression are high in individuals with chronic medical conditions such as coronary artery disease and congestive heart failure, and major depression is associated with significant increases in utilisation of health services, lost productivity and functional disability [6]. The National Service Framework for cardiac care in the UK therefore recommends screening patients for psychological illnesses [7].

Recent publications have raised awareness of depression as a possible negative predictive factor for overall cardiac outcome suggesting increased mortality in patients with depression after a myocardial infarction (post MI) [8]. It has been proposed that depression independently increases post MI mortality at six months [9]. A recent meta-analysis suggests that post MI depression is associated with a two to two-and-a-half fold increased risk of impaired cardiovascular outcome. But the association of depression with cardiac mortality was more pronounced in the studies before 1992 than in the more recent ones after 1992 [10]. Furthermore there is evidence to suggest that depressed older patients post MI are more likely to have co-morbidities, a four-fold increased risk of dying within the first twelve months after discharge and are less likely to be prescribed medication known to reduce post MI mortality. They also have greater difficulty following recommendations to reduce cardiac risk [11]. More recently Dickens et al found no association between depression and 1-year post MI mortality and concluded that the relationship is a complex one possibly being limited to depression immediately after MI [12]. This would suggest that the relationship between myocardial infarction and depression and anxiety disorders as well as their impact on mortality is still not entirely clear.

However, studies have shown that the use of antidepressants in depressed patients who experienced an acute MI might reduce subsequent cardiovascular morbidity and mortality [13,14]. The outcome of treated depression post MI appears to be better when Selective Serotonin Reuptake Inhibitors (SSRI) are used regarding cardiac as well as psychiatric outcome [13,14]. The standard components of Cognitive Behavioural Therapy (CBT) have also proven useful in treating co-morbid depression in post MI [15]. This proves that improved detection of mental disorder can lead to a profound benefit for cardiac patients as they are more likely to get appropriate treatment for their mental illness. In a large retrospective cohort study in general practice in the United Kingdom it has been shown that hazard ratios for coronary heart disease mortality in people with severe mental illness compared with controls is high at 3.22 for people between 18 to 49 years of age, 1.86 for those between 50 and 75, and 1.05 for those older than 75 years. This increased risk of death from coronary heart disease cannot be explained by taking antipsychotic medication, smoking, or social deprivation scores [16].

The main difficulty in assessing mental health of this group of patients is lack of time and in most instances lack of mental health assessment training and skills amongst physicians and other health professionals. One realistic possibility is to provide brief training to practitioners in the cardiac rehabilitation units to assess patients' mental health. We developed a computerised clinical mental health assessment tool called Global Mental Health Assessment Tool (GMHAT) to meet such needs in a general health setting.

Until recently cardiac rehabilitation services often did not routinely screen for psychiatric illness. More recently many services have started to use Hospital Anxiety and Depression Scale questionnaires in order to screen patients for depression and anxiety. This has particularly happened in the UK with the introduction of the National Service Framework for Cardiology, which puts an obligatory requirement on cardiac rehabilitation services to screen people for psychiatric diseases [7]. Screening questionnaires like HADS do not consider a broader spectrum of psychiatric disorders such as OCD, dementia, phobias and psychosis. This creates a requirement for a broader screening and diagnostic tool for mental health problems, which can be used in a cardiac rehabilitation population by non-psychiatrically trained people using a minimum amount of time.

## Aims and objectives

We carried out a study with the aim to a) assess the feasibility of nurses using a computer assisted diagnostic interview GMHAT/PC for patients attending Cardiac Rehabilitation Clinics and b) examine the level of agreement between the GMHAT/PC diagnosis and a Psychiatrist's clinical diagnosis. Prevalence of mental illness in this patient group was also measured.

## Methods

### Description of the Global Mental Health Assessment tool – Primary Care Version (GMHAT/PC)

The GMHAT/PC is a computer assisted interview that was developed to assist health professionals in making quick, convenient and comprehensive standardised mental health assessments. The GMHAT/PC has been developed by Evidence Based Centre, Cheshire and Wirral NHS Trust in Collaboration with University of Liverpool, UK. The GMHAT steering Group was founded in the 2001.

On the computer, the first screen captures patient demographics followed by a page with instructions to administer the tool and rate the symptoms. The following screens consist of questions about the patient's mental state, symptoms or problems. They start with two screening questions about every major symptom complex followed up by more questions if the screening questions are answered positively. The questions cover the following symptom areas: worries, anxiety and panic attacks, concentration, depressed mood, including suicidal risk, sleep; appetite, eating disorders, hypochondriasis, obsessions and compulsions, phobia; mania/hypomania, psychotic symptoms, disorientation, memory impairment, alcohol misuse, illegal drug misuse, personality problems and stressors. The questions proceed in a clinical order along a tree- branch structure. For each of the major clinical disorder there are key screening questions with cut off points, thus economising time. Most of the questions are based on the well established interview schedule GMS-AGECAT [17].

The diagnostic program takes account of the severity of symptoms. At the end of the interview a summary report of symptoms and their scores and a GMHAT/PC diagnosis is produced in a printable form. The main computer diagnosis is derived using a hierarchical model and designed around International Classification of Disorders (ICD) -10 (WHO 1992) [18]. It also generates alternative or additional diagnoses based on the presence of symptoms of other disorders. This tool takes on average 12 minutes to administer [19].

If required, the referral letter option prints out a letter of assessment with details of problems, symptoms with severity, and clinical diagnosis. In addition, it includes an assessment of risk of self-harm. The program contains management guidelines for all of these disorders if needed. GMHAT-pc is compatible with electronic patient records and hence makes communication across agencies easier.

The program is based on the Delphi (Borland) System and does not need any additional software programming support. A website for the tool http://www.gmhat.org has been constructed providing additional technical support.

This tool can be administered by doctors and nurses who do not need to be trained in mental health. No formal training is needed to administer this tool. However, before using the tool for the first time, it is preferable if the interviewers familiarise themselves with the GMHAT/PC by having a trial session

It has already been shown showed that the GMHAT/PC is a valid, reliable and feasible tool for the assessment of mental illness in people in primary care and secondary mental health settings [19,20]. The same group showed that the GMHAT/PC can assist nurses who were not trained in mental health to make accurate mental health assessments and diagnosis in patients [19]. The inter-rater reliability based on symptom scores and the correlation between the Hospital Anxiety and Depression scale (HADS) and GMHAT/PC depression scores has been found to be good [20].

We recruited patients through the Cardiac Rehabilitation Service at the Wrexham Maelor Hospital in North Wales. The Service is responsible for all referrals in the North East Wales area covering a population of around 250,000 people. Patients are primarily post-myocardial infarction but also post-angioplasty or valve repair surgery. They also include a small number of patients with heart failure. The only exclusion criterion for the service is significant cognitive impairment and mobility problems preventing them from active participation in the programme. Patients are usually seen between six to eight weeks following the event or surgery. Ethical approval was obtained through COREC (Central Office for Research Ethics Committee) and the local Ethics Committee.

Feasibility for the purpose of the study was defined as nurse and patient satisfaction and acceptance of GMHAT/PC and compatibility with clinical practice in terms of administration time. This data was gathered by the researcher asking for immediate verbal feedback from the subjects after the completion of the assessment. We asked the individuals administering the tool for its user-friendliness and if it prolonged the duration of the overall assessment. The feedback from the researchers administering the tool was gathered through a focus group. The time taken to administer GMHAT/PC is also considered as a measure of feasibility.

We asked all consecutive patients attending Wrexham Cardiac Rehabilitation Service to participate. Full informed consent was obtained from each patient agreed to participate by the consultant psychiatrist. Patients were not previously known to the nurses or the psychiatrist. GMHAT/PC assisted mental health assessments were carried out by the Cardiac Rehabilitation Nurses as part of their assessment for Cardiac rehabilitation programme. The cardiac nurses had no previous experience in mental health assessments. They had no training, but a two hour awareness session to familiarise them with the tool.

The key design element is a comparison of the assessment made by nurses generating a computerised mental health diagnosis with the diagnosis of an experienced consultant psychiatrist. We used Consultant Psychiatrists' diagnoses as a gold standard rather than a criterion standard. Although this is less desirable, in routine clinical care the diagnosis by a consultant psychiatrist is considered as an adequate gold standard. In order to avoid changes in the mental state of the patient over time the second (psychiatrist's) assessments were made immediately after the nurses' assessments. The psychiatrist made an ICD-10 criterion based clinical diagnosis through interviewing the patient. The assessments were done separately to avoid bias. The nurses and the consultant psychiatrist were both blind to the computerised diagnosis. Sensitivity and specificity data were obtained using mental illness diagnoses only, because physical diagnoses are not measured by the tool.

Patient demographics, cardiac diagnoses, nurse administered GMHAT/PC diagnosis, the psychiatrist's ICD-10 clinical diagnosis and time taken to administer the GMHAT/PC were recorded on the database. Prevalence data on all categories of mental illness were produced for this patient group. Patients were informed if a mental illness was detected by the consultant psychiatrist and a appropriate referrals for treatment were initiated.

### Statistical analysis

The kappa coefficient was used to determine the levels of agreement between the GMHAT/PC generated diagnoses and the Consultant Psychiatrists' diagnoses. We calculated the sensitivity and specificity of this tool to correctly identify the cases with and without mental illness compared to the Consultant diagnoses.

## Results

In total 124 consecutive patients were asked to participate in the study, of these 118 were recruited. Six patients did not participate: two refused, two were acutely unwell at the time and two for transport related reasons. Table 1 gives summary of patient demographics, 82 (69.5%) patients were male and 36 (30.5%) were female. The mean age (standard deviation) in the male group was 69.5 (10.5) years and for the female group it was 61.0 (12.3) years. The age range was 28–87 years. The mean time (standard deviation) taken for the administering GMHAT/PC by nurses in the male group was 13.3 (6.4) minutes and in the female group it was 14.9 (10.1) minutes. Though subjects had the option of discontinuation of the interview prematurely if it was uncomfortable or distressing, all patients completed the GMHAT/PC interview. Although there were more men in the study sample there was no statistically significant difference in age or time taken to complete GMHAT/PC.

**Table 1:** Patient demographics

	Males	Females	Combined
	82 (69.5%)	36 (30.5%)	118

Diagnosis of mental illness^2 ^n(%)	13 (15.9%)	13 (36.1%)	26 (22%)

Age mean (st.dev)^1^	61.9 (10.5) years	61.0 (12.3) years	61.6 (10.9) years
Range	28 – 85 years	33 – 87 years	28–87 years

Time mean (st.dev)^1^	13.3 (6.4) mins	14.9 (10.1) mins	13.9 (7.9) mins
range	2 – 33 mins	2–51 mins	2–51 mins

1 not significant (t-test) male's v females

2 Statistically significant difference (p = 0.03) chi-squared test males v females.

Of the 118 patients recruited to the study, 37 patients had a diagnosis of myocardial infarction, 30 had angina and 2 had heart failure. 30 patients were referred to the programme after a CABG (coronary artery bypass graft) and 19 following a PCI (percutaneous coronary intervention).

There was an excellent level of agreement between the nurses (GMHAT/PC) diagnosis and the psychiatrist diagnosis of mental illness with the correlation coefficient i.e. KAPPA 0.76 [95% CI (0.61, 0.91)]. There is good sensitivity of 0.73 95% CI (0.56, 0.90) and excellent specificity of 0.98 [95% CI (0.95, 1.00)] comparable with results from our earlier validation study [19]. Here we focus on feasibility, prevalence and usefulness of GMHAT/PC in this group of patients.

### Feasibility

Only two patients declined to participate in the study. None of the patients gave any negative feedback. Most of them expressed satisfaction that the nurse covered all aspect of their mental health using GMHAT/PC. The nurses who interviewed the patients found the GMHAT/PC user friendly and, as a consequence, have integrated it into their routine practice. With a mean of 14 minutes per GMHAT/PC interview it did not significantly lengthen the overall assessment time, especially since routine assessments would have incorporated some screening for mental illness.

### Prevalence of mental disorder

The overall prevalence of psychiatric disorder was 22%. Mental illness was significantly more common in women (36%) compared to men (16%), p = 0.03, skewing the results towards a lower overall percentage because men were over-represented in our sample. The prevalence of depression was 14.4% and that of anxiety 4.4%. The prevalence of other psychiatric disorders was psychosis 0.8%, dementia 0.8%, stress/adjustment disorder 0.8% and phobia 0.8%. Of 92 (78%) of patients identified as without mental illness, two of them were in fact patients with severe mental illness but who were currently in remission. One of them had a diagnosis of schizophrenia and the other severe depression.

Only 4 patients diagnosed with anxiety and depression combined were on antidepressant therapy and none of them received any psychological therapies, indicating a lack of provision of appropriate treatment.

Three patients were already known to secondary mental health services. Two of them had a diagnosis of depression and one had a diagnosis of schizophrenia. They were receiving appropriate treatment from the community mental health team. As a result of identification of mental disorders by using GMHAT/PC in this study one patient diagnosed with Psychosis (delusional disorder) and another diagnosed with depression was referred to Community Mental Health Teams for further assessment and treatment.

## Discussion

The findings of this study are encouraging and support the view that other health professionals, particularly nurses and possibly others can use the computer assisted GMHAT/PC to make valid assessments and diagnoses of mental disorder. GMHAT/PC in cardiac setting appears to have good sensitivity and excellent specificity. The mean duration of the interview was around 14 minutes and makes it feasible for routine assessments in general health settings as well as epidemiological studies. Both patients and nurses found the GMHAT/PC not only acceptable but also useful in making a quick and comprehensive mental health assessment.

Although there were relatively few patients with a diagnosis of psychosis in the study population, we have previously established GMHAT/PC as a valid and reliable tool for diagnosing patients with severe mental illness such as psychosis [20]. However, the prevalence of psychotic illness as opposed to the more common depression and anxiety in the general hospital setting is low and in keeping with the expected prevalence of approximately 1% in the general population.

Although 22% of patients were diagnosed with a mental illness only a small number of patients were on psychotropic treatment, none of them receiving formal psychotherapy. This reconfirms the finding that mental illness in cardiac patients is under-recognised and under-treated. The prevalence of mental illness in our study sample is comparable to other studies. As expected depression and anxiety disorders were common and also more common in women than men.

The findings of this study suggest high prevalence rates of co morbid psychiatric disorders as well as a broad spectrum of psychiatric disorders in cardiac rehabilitation outpatients. However, larger studies are needed in order to determine the true prevalence of these disorders in this cardiac patient group.

There was only one patient with a diagnosis of dementia. As patients with cognitive impairment were screened in pre-assessment stages and excluded from the cardiac rehabilitation service we are unable to comment on the use of the GMHAT/PC for the recognition and assessment of dementia. However, a similar study evaluating the validity and feasibility of GMHAT/PC in the elderly is under way and the preliminary findings are suggesting a good level of agreement, including in patients with dementia-like illnesses.

Until recently cardiac rehabilitation services did not routinely offer screening and assessment for psychiatric illnesses. However, since the implementation of the National Service Framework for cardiac patients many services have started to use screening tools like the HADS (Hospital Anxiety and Depression Scale) to identify patients with depression and anxiety. The dimensional structure and reliability of HADS is stable across medical settings and age groups. However, the correlation between age and HADS scores is small. The moderate Positive Predicative Value (PPV) of HADS suggests that HADS is best used as a screening questionnaire and not a "case identifier" for psychiatric disorder or depression [21]. We have also shown that there is good correlation between HADS scores and GMHAT/PC depression/anxiety scores [20]. HADS and other similar screening questionnaires are able to identify only a narrow range of mental health problems. Conditions like psychosis, phobia and OCD are less common than depression and anxiety in this population but nevertheless they need to be identified [4]. This creates a requirement for a broader screening and diagnostic tool for mental health problems like the GMHAT/PC, which can be used in cardiac rehabilitation populations by non-psychiatrically trained health professionals taking a minimum amount of time to administer. This should lessen the need for using multiple screening tools for various mental disorders. We acknowledge that diagnoses other than depression and anxiety in this sample are rare, but very significant (e.g. dementia or psychosis). We have already established in our previous studies that GMHAT/PC is a reliable assessment tool to identify a wide range of psychiatric diagnoses [19,20]. In addition to a primary diagnosis it can also generate additional diagnoses when symptoms of other disorders are present. The program contains management guidelines for all of these disorders if needed. It also offers other advantages of being compatible with electronic care records, the option of generating a referral to specialist teams and a risk assessment of self harm. Thus using GMHAT/PC in cardiac patients is greatly advantageous than other screening questionnaires like HADS.

In the UK various national service frameworks and policy guidelines have identified that mental health problems are significant co-morbidity factor in chronic physical illness as well as a serious hindrance to good outcome [22,23]. In combination with new ways of working it is clear that mental health diagnosis will have to be made increasingly by Health Professionals other than Psychiatrists [24]. Given that identification of mental health problems is vital to improve outcome in many chronic illnesses it is essential to improve the recognition rate. Studies on guideline dissemination have failed to demonstrate effectiveness in changing clinicians' behaviour, whereas more structured implementation strategies have produced some favourable results [25]. A number of case-finding tools are available but most of them are for depression and not many are used routinely [26]. The GMHAT/PC therefore helps not only in detection but also provides guidelines to help patients once mental health problems are identified. Similarly it also adds to the skills of Cardiac Nurses.

A structured interview schedule has its own advantages and disadvantages compared to an unstructured interview. There is evidence showing that in clinical practice an unstructured interview can miss essential items from both history and mental status examination [27]. There can be anxieties about the use of computer interviews as they might not be good at detecting non-verbal communication or patients may view them as impersonal. But evidence suggests that, generally, patients' satisfaction levels are positive when a computer is used in a psychiatric interview [28-32]. A short semi-structured computer interview like GMHAT/PC administered by health care professionals would combine the advantages of being both structured and comprehensive, whilst at the same time minimising the deficits, such as being impersonal and unable to pick non-verbal cues.

The GMHAT/PC computer-assisted diagnosis, which is based on symptom complexes present at the time of interview, is a useful aid in routine practice but is not intended to replace the clinical diagnosis, although the high level of agreement between the psychiatrist's clinical diagnosis and the computer-assisted diagnosis of the patients in the study is encouraging.

### Strengths and Limitations of this study

Consultant Psychiatrist and nurses doing the assessment in our study were blind to each other's diagnoses. The interviewer's had no knowledge of the patients before the assessment. We used Consultant Psychiatrists' diagnoses as a gold standard rather than a criterion standard. Although this is less desirable, in routine clinical care the diagnosis by a consultant psychiatrist is considered as an adequate gold standard. We did not attempt to examine the inter-rater reliability between the nurses who administered the GMHAT/PC. The relatively small number of subjects in this study limited from carrying out sub group analyses. There is no comparative group without detection of mental illness. The absence of follow up and outcomes did not allow proving changes in clinical management and outcomes.

### Implications for future research and practice

GMHAT-PC provides measurement of symptoms. It would be useful to see if this measurement is sensitive to change. If sensitive to measuring change, GMHAT/PC could also be used to monitor progress. The GMHAT full version has also been developed for a more comprehensive clinical assessment to provide diagnosis, risk assessments and outcome measures. It will also help health professionals to choose appropriate care pathways. Validation and Feasibility study of the full version GMHAT is already in progress. GMHAT/PC has been translated into various languages. Further studies are in progress in several countries to assess its validity and usefulness in different cultures. If this is successful GMHAT/PC could help health care professional across the world to make accurate mental health assessments in the elderly. This may help them to provide mental health services to a larger population.

## Abbreviations

GMHAT-PC: Global mental health assessment tool primary care version; HADS: Hospital Anxiety and Depression Scales; GMS: Geriatric Mental State Examination.

## Competing interests

The authors declare that they have no competing interests.

## Authors' contributions

MK and PL were local investigators involved in planning, recruitment, and organisation of the study, training and supporting cardiac nurses and preparing the manuscript. VS and JC developed the GMHAT-PC and were involved in designing the study. LL and MW interviewed and administered the instrument and were involved in designing the study.

## References

[B1] The world health report 2001 – Mental Health: New Understanding, New Hope 2001 World health Organization

[B2] Ginsburg K Co morbidity of PTSD and depression following myocardial infarction Journal of Affective Disorders 2006 94 1–3 135 43 16720050 10.1016/j.jad.2006.03.016

[B3] Faller H Störk S Schowalter M Steinbüchel T Wollner V Ertl G Angermann CE Depression and survival in chronic heart failure: Does gender play a role?, 03 August European Journal of Heart Failure 2007 9 10 1018 23 17660002 10.1016/j.ejheart.2007.06.011

[B4] Bankier B Januzzi JL Littman AB The High Prevalence of Multiple Psychiatric Disorders in Stable Outpatients With Coronary Heart Disease Psychosomatic Medicine 2004 66 645 650 15385686 10.1097/01.psy.0000138126.90551.62

[B5] Birket-Smith M Rasmussen A Screening for mental disorders in cardiology outpatients Nord J Psychiatry 2008 62 2 89 18569779 10.1080/08039480801983562

[B6] Egede L Major depression in individuals with chronic medical disorders: prevalence, correlates and association with health resource utilization, lost productivity and functional disability General Hospital Psychiatry 2007 29 5 409 16 17888807 10.1016/j.genhosppsych.2007.06.002

[B7] The Coronary Heart Disease National Service Framework: Building for the future – progress report for 2007 2008 Dept of Health

[B8] Frasure-Smith N Lespérance F Talajic M Depression and 18-Month Prognosis After Myocardial Infarction Circulation 1995 91 999 1005 7531624 10.1161/01.cir.91.4.999

[B9] Frasure-Smith N Lesperance F Talajic M Depression following myocardial infarction. Impact on 6-month survival JAMA 1993 270 1819 25 8411525

[B10] Van Melle JP de Jonge P Spijkerman TA Prognostic association of depression following myocardial infarction with mortality and cardiovascular events: a meta-analysis Psychosom Med 2004 66 814 22 15564344 10.1097/01.psy.0000146294.82810.9c

[B11] Romanelli J Fauerbach JA Bush DE Ziegelstein RC The significance of depression in older patients after myocardial infarction J Am Geriatr Soc 2002 50 817 22 12028166 10.1046/j.1532-5415.2002.50205.x

[B12] Dickens C McGowan L Percival C Douglas J Tomenson B Cotter L Heagerty A Creed F Association Between Depressive Episode Before First Myocardial Infarction and Worse Cardiac Failure Following Infarction Psychosomatics 2005 46 6 523 28 16288131 10.1176/appi.psy.46.6.523

[B13] Taylor CB Youngblood ME Catellier D Effects of Antidepressant Medication on Morbidity and Mortality in Depressed Patients After Myocardial Infarction Archives of General Psychiatry 2005 62 7 711 2 15997021 10.1001/archpsyc.62.7.792

[B14] Glassman AH O'Connor CM Califf RM Swedberg K Schwartz P Bigger JT Jr Krishnan KR van Zyl LT Swenson JR Finkel MS Landau C Shapiro PA Pepine CJ Mardekian J Harrison WM Barton D Mclvor M Sertraline treatment of major depression in patients with acute MI or unstable angina: Sertraline Antidepressant Heart Attack Randomized Trial JAMA 2002 288 701 9 12169073 10.1001/jama.288.6.701

[B15] Cowan Marie J Freedland Kenneth E Burg Matthew M Saab Patrice G Youngblood Marston E Cornell Carol E Powell Lynda H Czajkowski Susan M Predictors of Treatment Response for Depression and Inadequate Social Support – The ENRICHD Randomized Clinical Trial .psychotherapy and psychosomatics Psychother Psychosom 2008 77 27 37 18087205 10.1159/000110057

[B16] Osborn D Levy G Nazareth I Petersen I Islam A King MB Relative Risk of Cardiovascular and Cancer Mortality in People With Severe Mental Illness From the United Kingdom's General Practice Research Database Arch Gen Psychiatry 2007 64 2 242 249 17283292 10.1001/archpsyc.64.2.242

[B17] Copeland JR Dewey ME Griffiths-Jones HM A computerized psychiatric diagnostic system and case nomenclature for elderly subjects: GMS and AGECAT Psychol Med 1986 16 89 99 3515380 10.1017/s0033291700057779

[B18] ICD-10 Classification of mental and behavioural disorders Clinical description and diagnostic guidelines 1992 WHO, Geneva

[B19] Sharma Vk Lepping P Krishna M Durrani S Copeland JRM Mottram P Parhee R Quinn B Lane S Cummins A Can Nurses Make Accurate Mental Health Diagnosis Using GMHAT/PC? A Validity and Feasibility Study Br J Gen Pract 2008 58 551 411 6 18505618 10.3399/bjgp08X299218 PMC2418993

[B20] Sharma VK Lepping P Cummings AGP Copeland JRM Parhee R Mottram P The Global Mental Health Assessment Tool- Primary Care Version (GMHAT/PC). Development, reliability and validity World Psychiatry 2004 3 2 115 19 16633473 PMC1414685

[B21] Spinhoven P Ormel J Sloekers PP Kempen GI Speckens AE Van Hemert AM A validation study of the Hospital Anxiety and Depression Scale (HADS) in different groups of Dutch subjects Psychol Med 1997 27 2 363 70 9089829 10.1017/s0033291796004382

[B22] Department of Health National Service Framework for Mental Health, (HCS1999/222) London 1999

[B23] Department of Health Meeting the physical needs of individuals with mental health problems and the mental health needs of individuals cared for in general health sectors: Advice from the Standing Nursing and Midwifery Advisory Committee (SNMAC) 2005

[B24] Mental Health: New Ways of Working for Everyone-*Developing and sustaining a capable workforce* 2007 Department of Health

[B25] Croudace T Evans J Harrison G Sharp DJ Wilkinson E McCann G Impact of the ICD-10 Primary Health Care (PHC) diagnostic and management guidelines for mental disorders on detection and outcome in primary care. Cluster randomised controlled trial Br J Psychiatry 2003 182 20 30 12509314 10.1192/bjp.182.1.20

[B26] Shedler J Beck A Bensen S Practical Mental Health Assessment in Primary Care. Validity and Utility of the Quick PsychoDiagnostics Panel J Fam Pract 2000 49 7 614 621 10923571

[B27] Weitz WD Morgan DW Cuyden TE Robinson JA Toward a more efficient mental status examination. Free form or operationally defined Archives of general psychiatry 1973 28 2 215 18 4684287 10.1001/archpsyc.1973.01750320049008

[B28] Greist JH Gustafson DH Stauss FF Rowse GL Laughren TP Chiles JA A computer interview for suicide risk prediction American Journal of Psychiatry 1973 130 1327 32 4585280 10.1176/ajp.130.12.1327

[B29] Angle HV Ellinwood E A Psychiatric Assessment-Treatment-Outcome Information System: Evaluation with Computer Simulation Ellinwood Proc Annu Symp Comput Appl Med Care 1978 9 149 59

[B30] Carr AC Ghosh A Response of phobic patients to direct computer assessment The British Journal of Psychiatry 1983 142 60 65 6831131 10.1192/bjp.142.1.60

[B31] Petrie K Abell W Responses of parasuicides to a computerized interviewcomputers in Human Behavior 1994 10 415 418

[B32] Kobak KA Reynolds WM Greist JH Computerized and clinician assessment of depression and anxiety: Respondent evaluation and satisfaction Journal of Personality Assessment 1994 63 173 180 7932028 10.1207/s15327752jpa6301_14

